# Integrated single-cell multiomics reveals novel immune candidate markers for post-traumatic coagulopathy

**DOI:** 10.3389/fimmu.2022.1095657

**Published:** 2023-02-08

**Authors:** Ping Zheng, Ning Zhang, Dabin Ren, Cong Yu, Bin Zhao, Qingke Bai, Yisong Zhang, Wanju Sun

**Affiliations:** ^1^ Department of Neurosurgery, Shanghai Pudong New area People’s Hospital, Shanghai, China; ^2^ Key Laboratory, Shanghai Pudong New area People’s Hospital, Shanghai, China; ^3^ Department of Neurosurgery, Shanghai Fengxian Hospital, Shanghai, China; ^4^ Department of Neurosurgery, Second Hospital affiliated to Anhui Medical University, Shanghai, China; ^5^ Department of Neurology, Shanghai Pudong New area People’s Hospital, Shanghai, China; ^6^ Department of Emergency Medicine, Shanghai Pudong New area People’s Hospital, Shanghai, China

**Keywords:** single-cell, multi-omics, RNA-seq, TCR-seq, post-traumatic coagulopathy

## Abstract

**Introduction:**

Post-traumatic coagulopathy (PTC) is a critical pathology in traumatic brain injury (TBI), however, its potential mechanism is not clear. To explore this in peripheral samples, we integrated single cell RNA-sequencing and T cell repertoire (TCR)-sequencing across a cohort of patients with TBI.

**Methods:**

Clinical samples from patients with more brain severity demonstrated overexpression of T cell receptor–encoding genes and less TCR diversity.

**Results:**

By mapping TCR clonality, we found patients with PTC have less TCR clones, and the TCR clones are mainly distributed in cytotoxic effector CD8+T cell. In addition, the counts of CD8+ T cell and natural killer (NK) cells are associated with the coagulation parameter by WGCNA, and the granzyme and lectin-like receptor profiles are also decreased in the peripheral blood from TBI patients, suggesting that reduced peripheral CD8+ clonality and cytotoxic profiles may be involved in PTC after TBI.

**Conclusion:**

Our work systematically revealed the critical immune status in PTC patients at the single-cell level.

## 1 Introduction

Post-traumatic coagulopathy (PTC) is one of the critical pathophysiologies in a traumatic brain injury (TBI) population (1). Recent statistics show that approximately two-thirds of severe TBI patients have abnormal coagulation function on admission (2), and we previously show that coagulopathy and initial head computer tomography (CT) signs could be independent risk factors for progressive hemorrhagic injury (PHI) including epidural, subdural hematoma, brain contusion, subarachnoid hemorrhage, cranial fracture, and plasma D-dimer level (3, 4). The mechanisms of PTC include platelet dysfunction, endogenous anticoagulation, endothelial activation, abnormal fibrinogen (Fg), neuroinflammation, and hyperfibrinolysis (2, 5–7); however, up to now, the exact mechanism is unclear.

The key role of peripheral blood in TBI has been identified in our study and in other studies, and several biomarkers in peripheral blood mononuclear cells (PBMCs) are related to neurodegenerative diseases (8). In this study, we collected PBMCs from 12 TBI patients and four healthy controls for single-cell RNA sequencing (scRNA-seq) and TCR sequencing. The clonal expansion of CD8+ T cells in TBI and PTC progression was identified in TBI and PTC, which were further validated using fluorescence-activated cell sorting (FACS). Our finding might be useful for the development of immune cell-targeted therapy in PTC.

## 2 Experimental section

### 2.1 Participants

Four normal individuals and 12 TBI patients (4 with mild TBI, 4 with moderate TBI, and 4 with severe TBI) were enrolled. PBMCs from 11 TBI patients were further collected for validated FACS and correlation analysis with the patients’ GCS scores with randomization. The inclusion criteria are as follows: a clinical diagnosis of TBI, an age range of 18–60 years, and no previous history of brain, liver, or hematologic disorders. Patients were excluded if they met the following criteria: active systemic infection or multiple injury, use of immunosuppressants, and other serious coagulopathy not related to trauma.

The consent form was obtained from patients’ family members. Patients’ characteristics are summarized in **Tables S1** and **S2**. There were no subjects dropped out from the study. No female patients were on progestin. This project was approved by the Ethics Committee of Shanghai Pudong New Area People’s Hospital (approval number 2021-K02) and was conducted in accordance with the Declaration of Helsinki.

### 2.2 Head CT imaging

The diagnosis of PHI was previously reported(3,4).

### 2.3 Post-traumatic coagulopathy definition

The coagulation parameters include the prothrombin time (PT), international normalized ratio (INR), activated partial thromboplasting time (APTT), Fg, and platelet count (Plt). The PTC is defined as abnormal value of PT, INR, APTT, Fg, or Plt(3,4). Detailed information of coagulation parameters on these patients is shown in **Table S3**.

### 2.4 Microarray information

The Agilent Human lncRNA Microarray 2019 (4*180k, Design ID:086188) was used here. The RNA extraction and quantification method was previously reported.

### 2.5 ScRNA-seq method and TCR-seq

The exact procedure for scRNA-seq and TCR-seq for PBMCs could be obtained from the 10X website. Fresh blood samples from 12 TBI patients (Se1–4, Mo1–4, and Mi1–4) and four age-matched healthy controls (Co1–4) were collected and PBMCs were isolated with the assay. Single-cell 5′ and TCR V(D)J libraries were constructed according to the 10X Chromium Immune Profiling protocol. Briefly, CD3+ T cells (isolated with FACS) were loaded onto Chromium Controller (10X Genomics, USA) with a V(D)J Kit. Each single-cell 5’ and V(D)J library was sequenced with the Illumina Novaseq 6000.

### 2.6 Cell quality control

We carried out data quality control. We captured cells with less than 10% of mitochondrial genes, with the total number of genes ranging from 200 to 6,000 and were expressed in at least three cells. Doublets were removed with the DoubletFinder package with a doublet rate <5%. The number of highly variable genes was set at 3,000. These two samples were integrated through SCT correction. Then, both the tSNE and uMAP methods were used to reduce the dimension of data.

### 2.7 Fluorescence-activated cell sorting

PBMCs from 11 TBI patients were isolated by FACS to further validate the results of scRNA-seq. The exact procedure was previously reported (9).

### 2.8 Cell–cell interaction

Using scRNA-seq data, taking the gene expression data of cell subsets as the research object, with the help of the ligand–receptor database, the ligand and receptor information in cells can be obtained using cellphoneDB (10), and the signal communication relationship between cells can be obtained, to elucidate the complexity, diversity, and dynamics of cell-to-cell communication in a wide range of biological processes.

### 2.9 RNA velocity assessment

RNA velocity is the testing rate of change in the abundance of mRNA molecules in a cell (11). Previous studies have demonstrated that RNA velocity can be estimated from unspliced and spliced mRNA abundance, which can predict the future state of individual cells on a timescale of hours (11). RNA velocity can reveal the dynamics of single-cell gene expression on timescales that match developmental, regeneration, and response processes in humans and other mammals. We employed the velocyto algorithm to estimate changes in RNA abundance over time by calculating the ratio of intracellular mRNA before and after splicing, and infer the next possible differentiation direction of cells.

### 2.10 Weighted co-expression network analysis

Weighted co-expression network analysis (WGCNA) is a computational method to describe co-expressed genes between different groups. It can identify marker genes based on the non-orientation analysis between the gene set and phenotypes. Here, WGCNA was used to locate the gene modules related to coagulation dysfunction in TBI.

### 2.11 Statistical analysis

Statistical analysis was performed using GraphPad Prism software (v8.1, GraphPad Software Inc., San Diego, CA, USA). The data were analyzed by those who were blinded to patients’ grouping. No power analysis was required as we had four subjects in each group. The correlation among the number of T cells, the expression levels of genes, and GCS scores (the severity of TBI) was analyzed using the Pearson correlation. Any difference with a *p*-value < 0.05 was considered significant. The exact method for analysis was mentioned in essential parts.

## 3 Results

### 3.1 Single-cell transcriptional landscape of PBMCs from TBI patients

The schematic plot of the study is graphed in **Figure S1**. Head CT scans showed progressive brain contusions in PTC patients (**Figures S2A, B**) compared to nPTC individual (**Figures S2C, D**). The chipset results for peripheral blood of TBI patients showed that the hallmark of coagulation was among the enriched pathways (**Figures S2E, F**). The KEGG analysis also showed that coagulation cascades were activated in TBI, while the antigen processing and presentation were deactivated in TBI (**Figure S2G**). This leads us to investigate the role of immune system in coagulation after TBI. When we investigated the coagulation parameter and immune profiles, we found the increased expression of CD8+ T cells in PBMCs of patients with more severe brain injury (**Figure S2H**) and the increased count of CD8+ T cells was positively associated with the increased Fg value; meanwhile, the counts of NK cells had a negative correlation with the PT and INR value.

**Figure 1:**
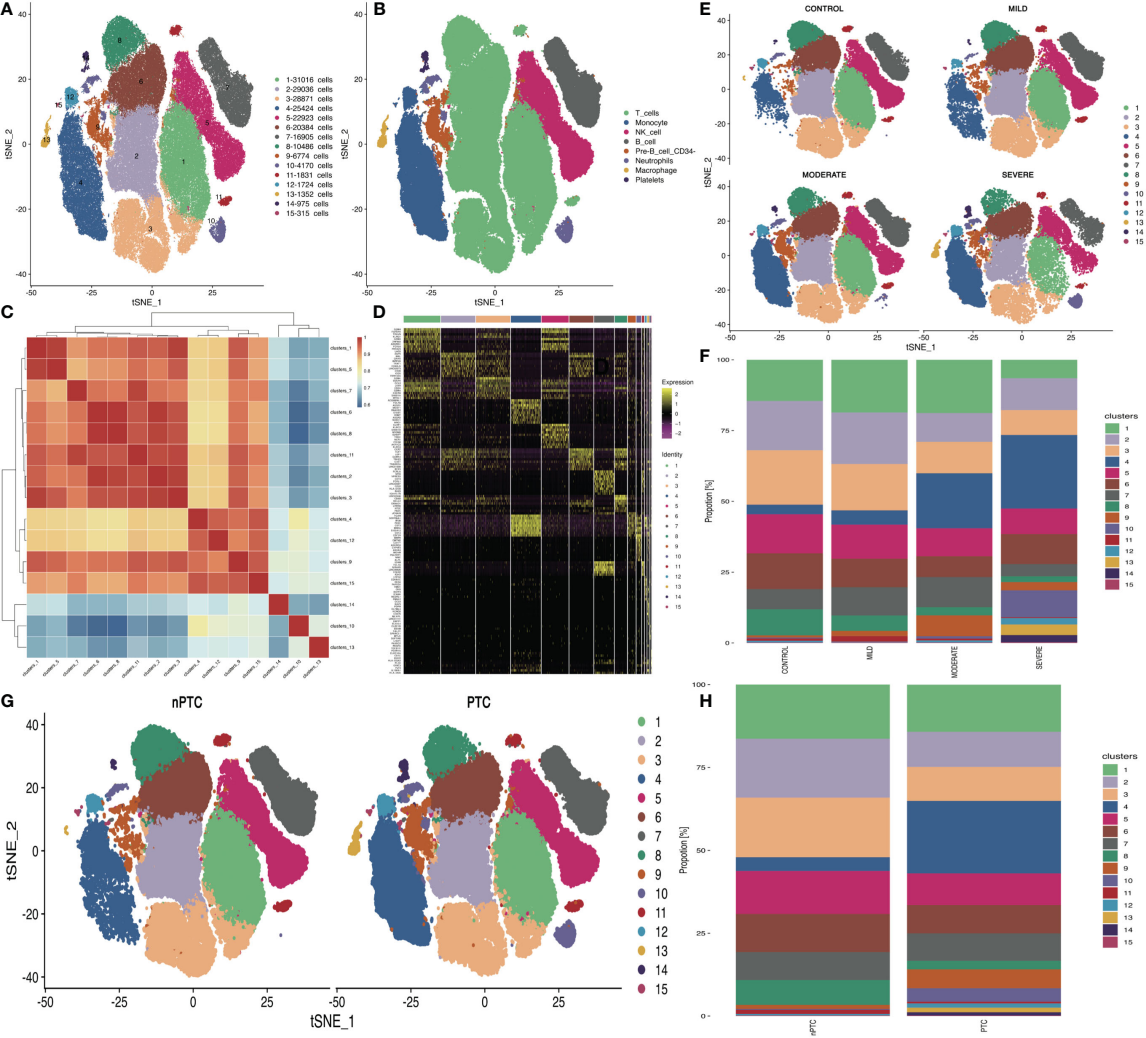
The differences in cell subsets of PBMCs in TBI patients. **(A)** t-SNE plot shows the 15 cell subsets in all PBMC samples from 12 TBI patients and 4 healthy controls. **(B)** Cell annotations in eight clusters. **(C)** Correlations of 15 cell subsets are analyzed with Pearson method for the mean expression of genes between each cluster to investigate the similarity between each cell cluster. The *X*- and *Y*-axis is each cell cluster. The red color indicates the higher correlation similarity, while the blue color suggests the lower correlation. **(D)** Top 10 cell markers in each cluster. **(E)** The t-SNE distribution of 15 cell subsets in different TBI groups. **(F)** The bar plot of 15 cell subsets’ distribution in different TBI groups. **(G)** The t-SNE distribution of 15 cell subsets’ distribution in nPTC and PTC groups. **(H)** The bar plot of 15 cell subsets in nPTC and PTC groups.

To explore the peripheral immune status in TBI, we collected PBMCs from TBI patients for scRNA-seq based on 10x Genomics. Cells were subjected to t-distributed stochastic neighbor embedding (t-SNE) analysis, and 15 clusters in four groups were obtained (**Figure 1A**). The cell annotation identifies eight clusters, namely, T cells, monocytes, NK cells, B cells, pre-B cells, neutrophils, macrophages, and platelets (**Figure 1B**). About 1,300 highly variable genes were identified in 16 samples, and less genes (982) were found in mi2 (a sample from a mild TBI patient) (**Table S2**). The pairwise correlation between intergroup variability ranges from 0.8101 to 0.9496 (**Figure 1C**).

Through the clustering analysis, the top 10 cell markers in each cluster were analyzed in two dimensions with Monocle2 (**Figure 1D**). Then, the quantification data showed high densities of T cells and NK cells in each sample (**Figures 1E, F**). In the PBMCs of PTC patients, the percentage of T and NK cells was obviously reduced compared with those of nPTC groups as well (**Figures 1G, H**).

The differentially expressed genes (DEGs) between different TBI groups and the control are listed in **Figures S3A–C**. The DEGs between PTC and nPTC groups are listed in **Figure S3D**. Next, KEGG analysis of DEGs identified by scRNA-seq was mainly enriched in the immune system, infectious disease, and signal transduction (**Figures S3E–G**). The similar enriched pathways were identified between the PTC and the nPTC group (**Figure S3H**). Then, the bubble plot shows that the upregulated genes identified by scRNA-seq were mainly involved in ko04620: Toll-like receptor signaling pathway; ko04612: antigen processing and presentation; and ko04610: complement and coagulation cascades (**Figures S3I, J**).

### 3.2 Decreased TCR diversity in PBMCs from TBI patients

**Figure 2:**
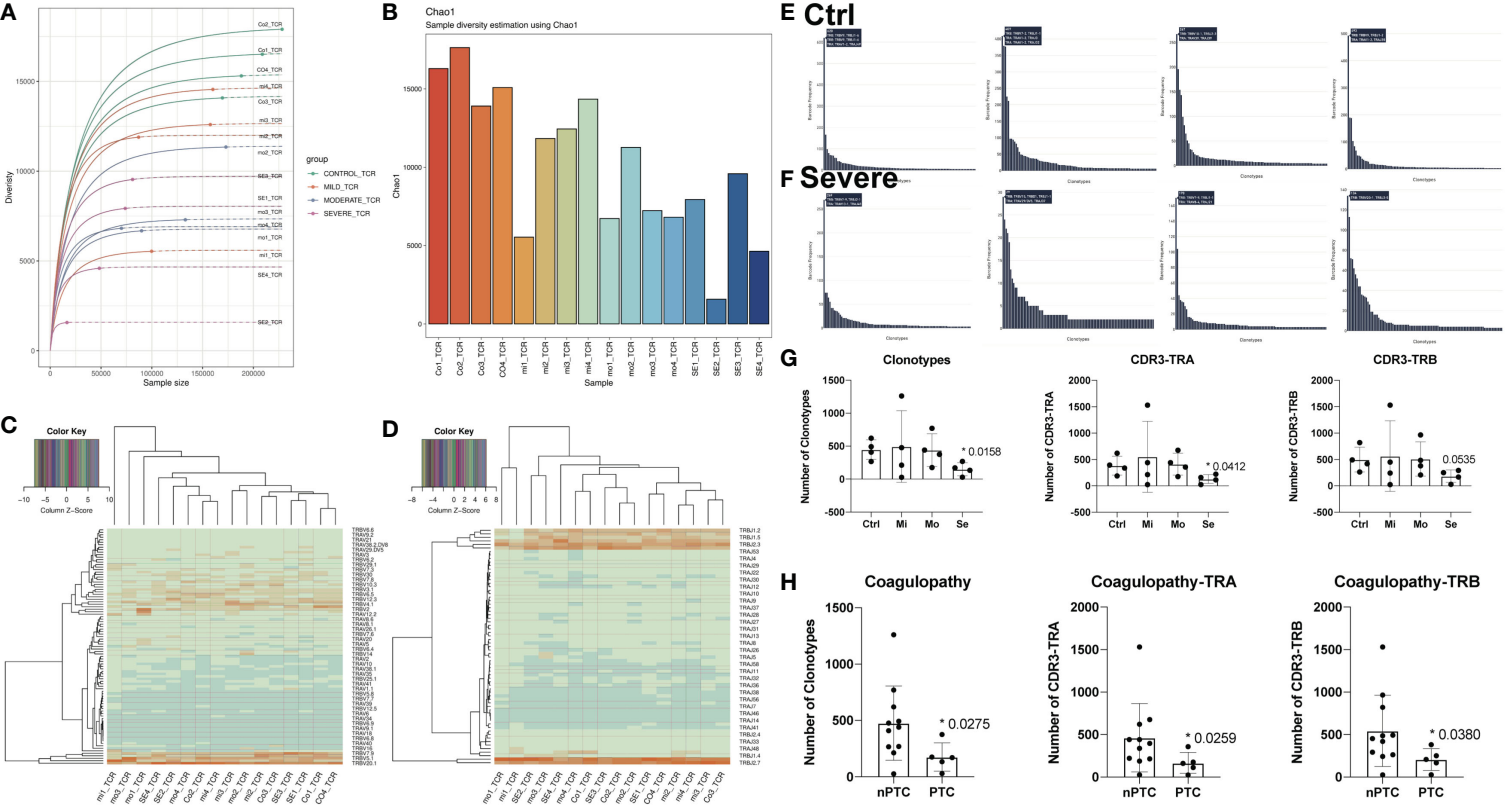
The differences of T-cell clonity in TBI patients. **(A)** Clonotype richness of TCR in different samples. **(B)** Chao1 index of TCR in different samples. **(C, D)** The relative expression of V and J genes in different groups. **(E, F)** The number of clonotypes of TCR genes in the control and severe group. **(G)** The quantification of clonotypes CD3-TRA and CD3-TRB in different TBI groups. **(H)** The quantification of clonotypes CD3-TRA and CD3-TRB between nPTC and PTC group.

To further accurately identify the peripheral immune function in TBI, we performed TCR-seq for collected PBMCs from TBI patients based on 10x Genomics again. The dilution curve of the sample list in **Figure 2A** shows the dependence between clonotype diversity and clonotype size. In theory, if the sequencing depth is large enough, ideally, the curve will eventually tend to be horizontal. Here, we can directly show the difference in clonotype richness between samples and can also find that the diversity of TCR repertoire was largely reduced with the severity of TBI. Again, the Chao1 index also decreased in the severe TBI group, indicating that severe TBI disrupts the diversity of T cells (**Figure 2B**). Furthermore, TCR V–J gene combinations were conserved in the TBI groups and the control group (**Figure S4**), and the relative expression of V genes (**Figure 2C**) and J genes (**Figure 2D**) in different groups was also conserved. These results suggested that TCR genes were conserved among groups. As the diversity of T cells decreased in TBI, we further investigated the number of clonotypes of TCR genes and found that it was obviously decreased in the severe TBI groups (**Figures 2E, F**) regarding the number of clonotypes, number of CDR3-TRA, and number of CDR3-TRB (**Figure 2G**). The difference also occurred between nPTC and PTC groups (**Figure 2H**). This indicates that CDR3 genes might be a biomarker for TBI and also involved in PTC.

### 3.3 Expanded CD8+ T cells in TBI patients

Therefore, we further looked at the distribution of different CDR3 lengths and found that the CDR3 length increased in the severe TBI group and the distribution of CDR3 length shows a symmetry style in the control group while it demonstrates a bias distribution (**Figure S5**). These findings indicate that the severe TBI group might have T-cell expansion, which leads us to further explore the leading role of CD4+ T and CD8+ T cells in TBI.

**Figure 3:**
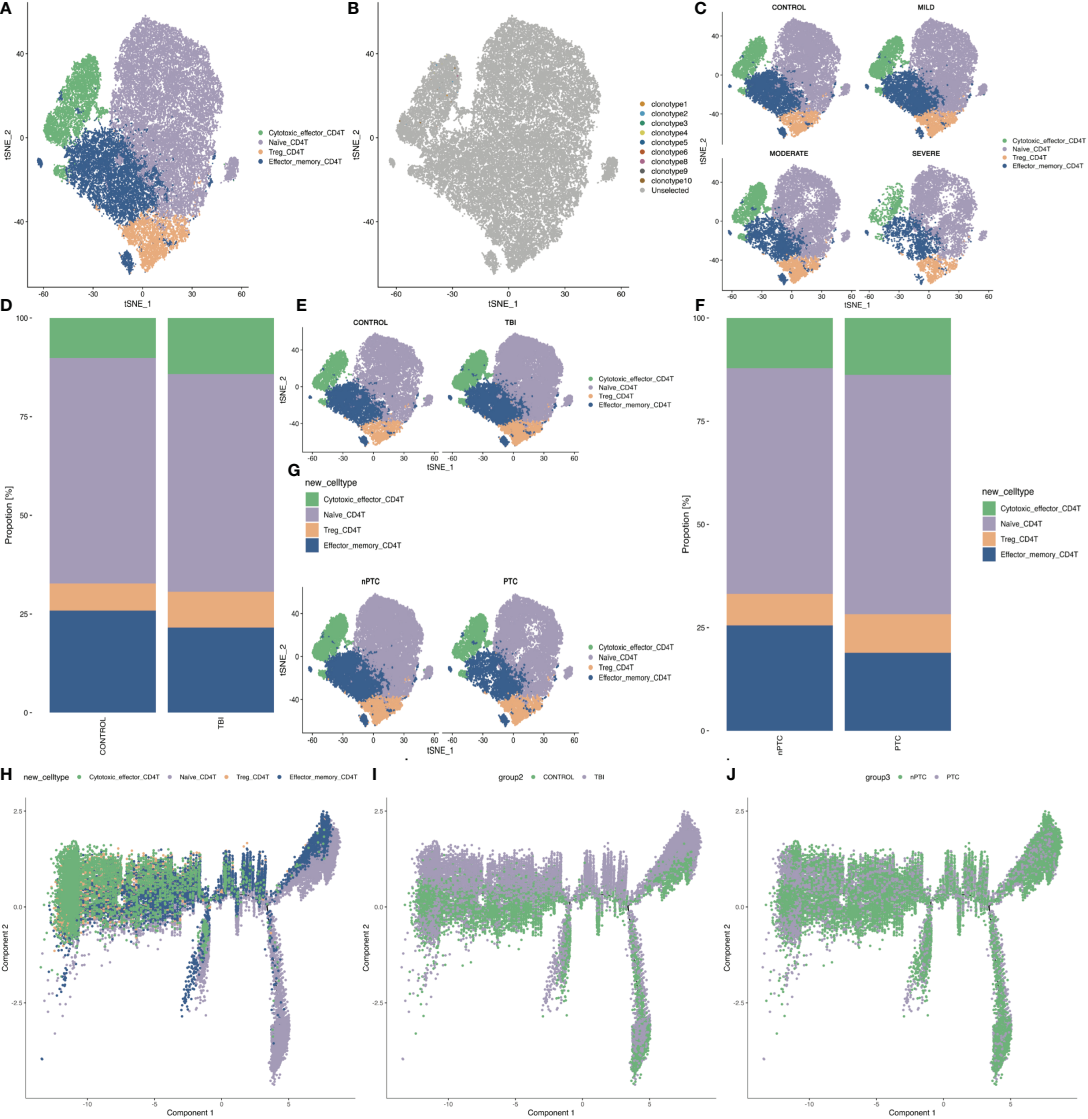
The subpopulations of CD4+ T cells of PBMCs in TBI patients. **(A)** The four clusters of CD4+ cells in t-SNE map. **(B)** The TCR clone in each CD4+ cell cluster. **(C)** The t-SNE map shows the different distribution of four clusters in control, mild, moderate, and severe TBI. **(D, E)** t-SNE map shows the four CD4+ cell subsets between TBI and control and quantification of the proportion of cells in each group. **(F, G)** t-SNE map shows the four CD4+ cell subsets between nPTC and PTC groups and quantification of the proportion of cells in each group. **(H–J)** Pseudo-time analysis for CD4 subsets between control vs. TBI and nPTC vs. PTC group.

We first looked at the subpopulations of CD4+ T cells of PBMC in TBI patients and identified four clusters: Naïve CD4 T, Treg, Effector_memory CD4 T, and cytotoxic effector CD4 T (**Figure 3A**). There are very few TCR clones in CD4+ T cells, and they are sparsely distributed in cytotoxic effector CD4 T (**Figure 3B**). Next, the t-SNE map shows the different distribution of these clusters in control and TBI groups. We found that the number of effector_memory CD4 T cells decreased with the severity of TBI (**Figures 3C–E**). In addition, the number of effector_memory CD4 T cells was also reduced in the PTC group compared to the nPTC group (**Figures 3F, G**). Pseudo-time analysis for CD4 subsets shows that the Naïve CD4 T and effector_memory CD4 T cells are mainly distributed in control and nPTC groups, while the cytotoxic_effector CD4 T and Tregs are dominant in TBI and PTC groups, which also have a trend from Naïve CD4 T to cytotoxic_effector CD4 T and Tregs (**Figures 3H–J**). The molecular markers of cytotoxic_effector CD4 T, Naïve CD4 T, Treg_CD4 T, and Effector_memory_CD4 T are listed in **Figures S6A–E**.

**Figure 4:**
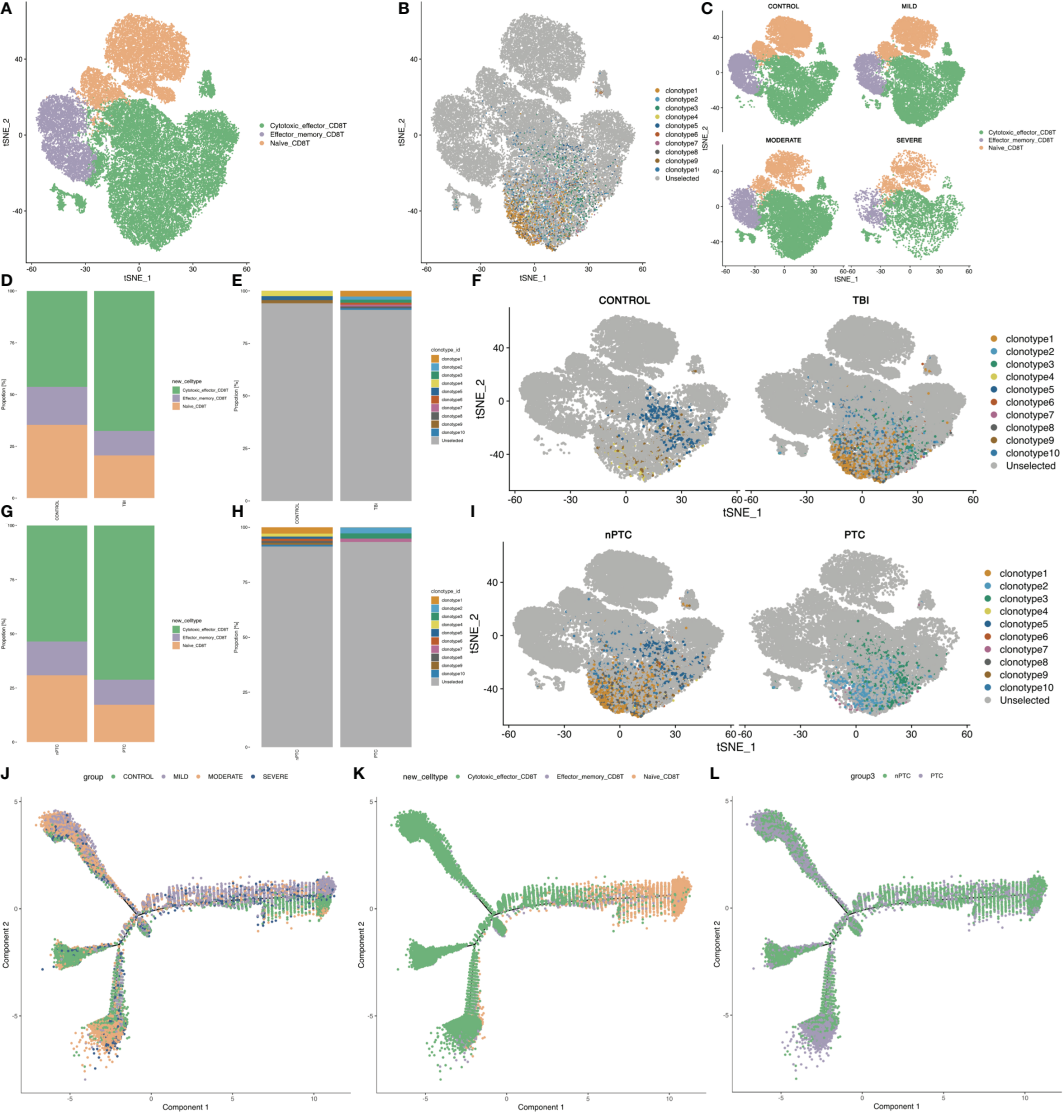
The subpopulations of CD8+ T cells and CD8 T clone of PBMCs in TBI patients. **(A)** The three clusters of CD8+ cells in the t-SNE map. **(B)** The TCR clone in each CD8+ cell cluster. **(C)** The t-SNE map shows the different distributions of three clusters in control, mild, moderate, and severe TBI. **(D–F)** t-SNE map shows the clone expansion between TBI and control and quantification of the proportion of TCR clonotype in each group. **(G–I)** t-SNE map shows the clone expansion between nPTC and PTC groups; and quantification of the proportion of TCR clonotype in each group. **(J–L)** Pseudo-time analysis for CD8 subsets between control vs. TBI and nPTC vs. PTC group.

Next, we investigated the subpopulations of CD8+ T cells of PBMC in TBI patients and identified three clusters: Naïve CD8 T, Effector_memory CD8 T, and Cytotoxic effector CD8 T (**Figure 4A**). The TCR clone is mainly distributed in cytotoxic effector CD8 T (**Figure 4B**). Next, the t-SNE map shows the different distribution of these clusters in control and TBI groups. We found that the quantity of cytotoxic effector CD8 T cells increased with the severity of TBI (**Figures 4C–F**). In addition, the clonotypes of cytotoxic effector CD8 T cells were also increased in the PTC group compared to the nPTC group, and the dominant clonotypes shifted between these two groups as well (**Figures 4G–I**). Pseudo-time analysis for CD8 subsets shows that the Naïve CD8 T and effector_memory CD8 T cells are mainly distributed in control and nPTC groups, while the cytotoxic_effector CD8 T is dominant in TBI and PTC groups, which also have a trend from Naïve CD8 T to cytotoxic_effector CD8 T (**Figures 4J–L**). The molecular markers of cytotoxic_effector CD8T, Effector_memory_CD8 T, and Naïve CD8T are listed in **Figures S7A–D**. These findings indicate the clonal expansion of CD8 T cells in the TBI, which might be involved in the PTC.

### 3.4 Cell–cell interaction in TBI patients

As the number of TCR clonity in TBI decreased, we further investigated the cell–cell interaction in the scRNA-seq data and found the cell–cell connection reduced in both TBI (**Figure S8**) and PTC compared to the control and nPTC groups (**Figure S9**); in particular, the interaction between T cells and other cell types decreased obviously in the PTC group (**Figures S9C, E**). Consistently, the RNA velocity map showed that the T-cell subset differentiation was also decreased in the TBI group compared to the control group (**Figures S8G, H**). The decreased differentiation ability of T cells also occurred in the PTC group compared to the nPTC group (**Figures S9G, H**).

### 3.5 Validation of immune cell proportion in peripheral blood by FACS

**Figure 5:**
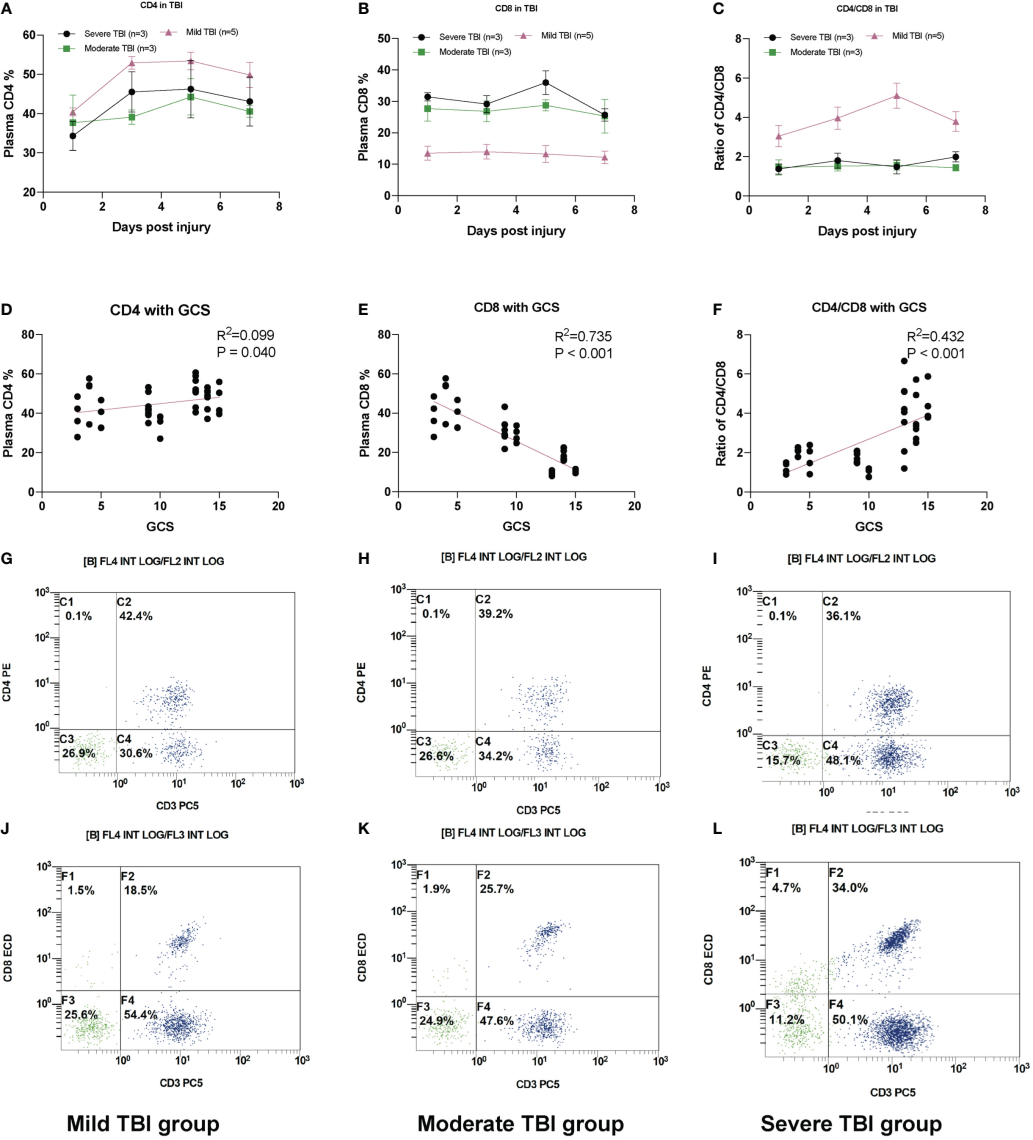
Plasma CD8+ T-cell increases in TBI. **(A)** The relative plasma CD4 percentage (reflected by CD3+ CD4+) in TBI groups. **(B)** The relative plasma CD8 percentage (reflected by CD3+ CD8+) in TBI groups. **(C)** The ratio of CD4/CD8 in TBI groups. **(D)** The scatter plot of plasma CD4 T% with GCS. **(E)** The scatter plot of plasma CD8 T% with GCS. **(F)** The scatter plot of CD4/CD8 with GCS. **(G)** The representative FACS image of CD4 (reflected by CD3+ CD4+) in severe TBI groups. **(H)** Moderate TBI and **(I)** mild TBI. The representative FACS image of CD8 (reflected by CD3+ CD8+) in **(J)** mild TBI groups. **(K)** Moderate TBI and **(L)** severe TBI. *N* = 3–5 in each group.

The immune system impairment has been previously reported in severe infection and neurodegenerative disease, both indicating the inverted ratio of CD4 T/CD8 T cells. Here, we also found this trend from our scRNA-seq results and further validated in our FACS analysis (**Figure 5**) and previous report (12). The plasma percentage of CD4+ T cells and ratio of CD4/CD8 decreased with the severity of TBI, while the ratio of CD8+ T cells increased. Both the CD4 expression and CD4/CD8 ratio show a positive correlation with the GCS score, while the CD8 expression has a negative correlation with the GCS score (**Figures 5A–G**). The representative FACS images of CD4 and CD8 in mild, moderate, and severe TBI groups are demonstrated in **Figures 5H–M**.

### 3.6 The cytotoxic profiles decrease in PTC groups

**Figure 6:**
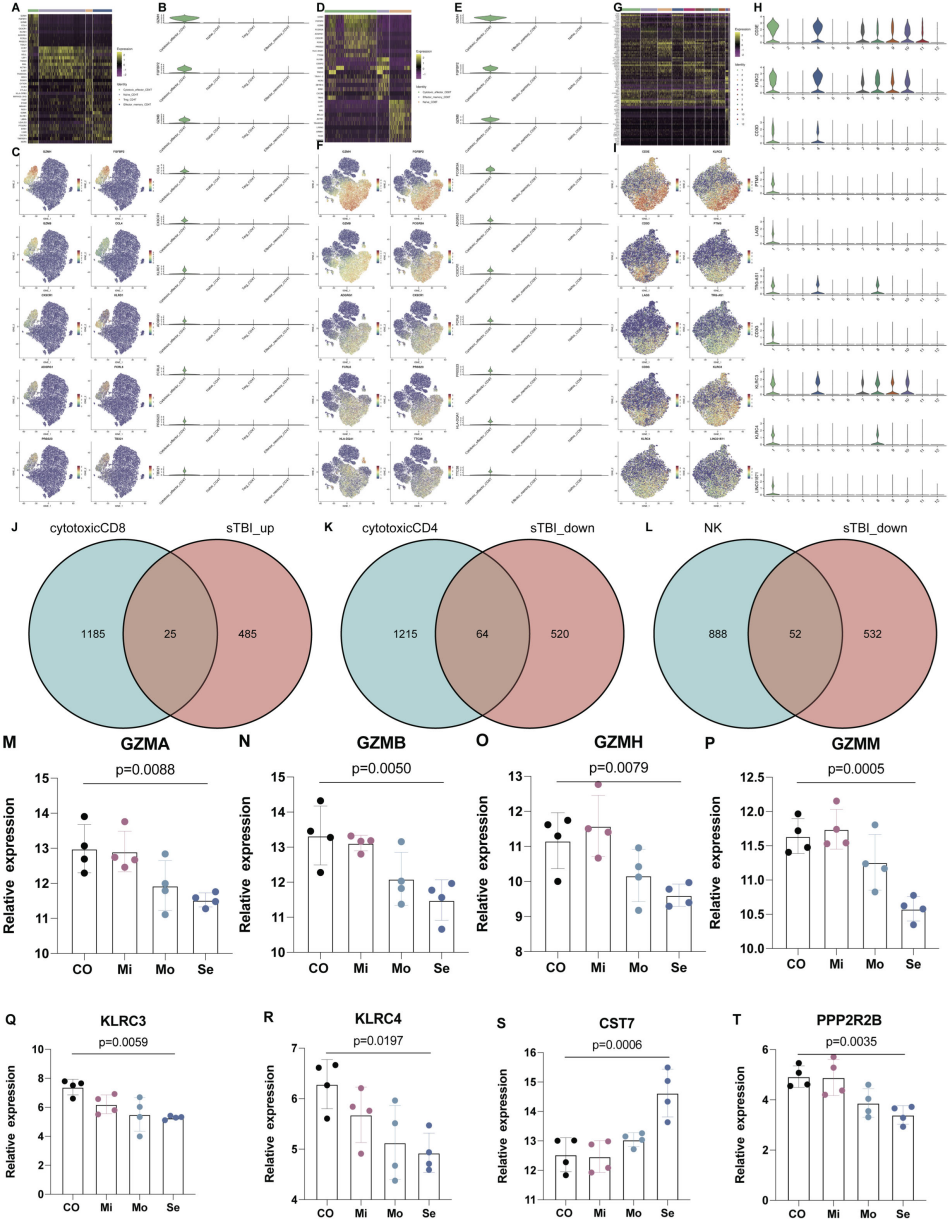
The marker genes for subtypes of CD4, CD8, and NK cells. **(A)** Heatmap shows the DEGs in Naïve CD4 T, Treg, Effector_memory CD4 T, and cytotoxic effector CD4 T. **(B, C)** Marker genes for cytotoxic effector CD4 T. **(D)** Heatmap shows the DEGs in Naïve CD8 T, Effector_memory CD8 T and Cytotoxic effector CD8 T. **(E, F)** Marker genes for cytotoxic effector CD8 T. **(G)** Heatmap shows the DEGs in NK cell subsets. **(H, I)** Marker genes for NK cell subsets. **(J–L)** Intersected marker genes of brain injury in cytotoxic CD8+, CD4+, and NK cells. **(M–P)** The expression of cytotoxic markers (granzyme **A, B, H, M**) in peripheral blood of TBI patients. **(Q, R)** The expression of c-type lectin receptors in peripheral blood of TBI patients. **(S)** The expression of CST7 as a marker of cytotoxic CD8 T cells. **(T)** The expression of PPP2R2B as a marker of NK cells. *n* = 4 in each group and one-way ANOVA is applied.

In addition, marker genes in cytotoxic CD8+ T, CD4+ T, and NK cells were also identified in PTC (**Figure 6**). The marker genes for different subsets of cytotoxic CD4+ T, CD8+ T, and NK cells were identified in **Figures 6A–I** for cytotoxic_effector CD4 T, cytotoxic_effector CD8 T, and NK cells. Furthermore, the intersecting marker genes of these subsets are listed (**Figures 6J–L**), and the cytotoxic profile of both cells that was validated in the plasma chipset data from 12 TBI patients and four healthy controls shows that the expression of cytotoxic markers (granzymes A, B, H, and M) and c-type lectin receptors (**Figures 6M–P**) and a representative NK cell marker (PPP2R2B) was also reduced with more severity in TBI (**Figures 6Q–T**), while the expression of a representative marker for cytotoxic CD8 T cells, CST7, increased with the severity of TBI (**Figure 6S**).

## 4 Discussion

In this study, differentially expressed cell subpopulations were screened in PBMCs of TBI patients by scRNA-seq analysis; CD8+ T cells represented a crucial cell subset in the progression of TBI, as verified through FACS in TBI patients. The mechanism of PTC has been a challenging undertaking. Coagulation characterization with peripheral blood has been regarded as the standard diagnosis for PTC, but this method is a traditional clinical test, which has limited extensibility in terms of treatment target.

Immune cells, particularly lymphocytes, may be involved in the pathogenesis of PTC. The scRNA-seq results in this work provide a detailed view of PBMCs in normal and TBI patients, in which the top eight cell subsets were T cells, monocytes, NK cells, B cells, pre-B cells, neutrophils, macrophages, and platelets, and the cell difference in each cluster can be clearly seen (**Figure 1D**). Furthermore, while studying signaling pathways, KEGG analysis revealed that the upregulated genes in PBMCs were mainly enriched in the following pathways: antigen processing, presentation, and coagulation, while the similar enriched pathways were identified between the PTC and nPTC group: Toll-like receptor signaling pathway, antigen processing and presentation, and complement and coagulation cascades.

In addition, cytotoxic profiling genes in CD8+ T, CD4+ T, and NK cells were identified as marker genes (**Figure 6**). The cytotoxic profile of all these cells was validated in the plasma chipset data from 12 TBI patients and four healthy controls and shows that the expression of cytotoxic markers (granzymes A, B, H, and M) and c-type lectin receptors is reduced with more severity in TBI (**Figures 6J–O**). Immediately, the investigation value of CD8+ T cells in TBI stands out from other cell subsets. Afterward, the results were continuously expanded in 11 TBI patients through FACS, and CD8+ T cells were found to be specifically increased in PBMCs of severe TBI patients compared to mild TBI groups, and the increased number of CD8+ T cells was closely associated with TBI severity. A similar study showed that the percentage of CD8+ T cells in the lymphocyte subsets of TBI were increased compared with healthy people (12), which was consistent with our study.

To verify the role of CD8+ T cells in TBI progression, we further exploited TCR-seq for collected PBMCs from TBI patients based on 10x Genomics. First, we found that the diversity of the TCR repertoire was largely reduced with the severity of TBI. Correspondingly, the number of clonotypes of TCR genes, specifically the number of CDR3-TRA and CDR3-TRB, was also decreased in the severe TBI groups. This indicates that CDR3 genes might be a biomarker for TBI and also involved in PTC. However, when we further looked at the distribution of different CDR3 lengths, we found that the CDR3 length increased in the severe TBI group. In particular, we found that the amount of effector_memory CD4+ T cells decreased and the number of cytotoxic effector CD8+ T cells increased with the severity of TBI. In addition, the clonotypes of cytotoxic effector CD8+ T cells were also increased in the PTC group compared to the nPTC group. These findings indicate the clonal expansion of CD8+ T cells in TBI, which might be involved in the PTC. Functionally, the cell–cell interaction was also reduced in both TBI and PTC compared to the control and nPTC group; in particular, the interaction between T cells and other cell types decreased obviously in the PTC group. This is consistent with the RNA velocity map showing the reduced T-cell subset differentiation in TBI and PTC groups. Furthermore, the cytotoxic profiles in PBMC obviously decreased in the TBI and PTC groups, which is consistent with previous studies (13). All these findings suggest that CD8+ T cells might be involved in the PTC.

Traditionally, it would be difficult to link peripheral immune status to coagulation function. Consistent with our observation, one cellular therapy study of 100 patients with hematologic malignancies have coagulation disorders after chimeric antigen receptor (CAR)-T cell therapy, which demonstrated that imbalanced T-cell subsets may initiate the coagulopathy characterized by prolonged PT, APTT, INR, D-dimer, and decreased Fg (14). Meanwhile, patients accepting CD19 CAR-T cell therapy also demonstrate impaired coagulation function with severe cytokine release syndrome (15, 16). In addition, Johnsrud et al. also proposed that CAR-T cell therapy may trigger neurotoxicity as well (16). Accordingly, expanded CD8 T cells with reduced clonal diversity were identified in the early stage of TBI and might be involved in the mechanisms of PTC. Recently, immunity, endothelial injury, and complement-induced coagulopathy in COVID-19 have been the topic of many investigations, some of which consistently revealed that peripheral CD8+ T cells from patients with COVID-19 express high levels of exhaustion markers, including programmed cell death protein 1 (PD1) and T-cell immunoglobulin mucin-3 (TIM3) (17). However, the cytotoxic activity of CD8^+^ T cells in COVID-19 remains elusive, which are assessed in three ongoing clinical trials (17). Cytotoxic CD8^+^ T cells in TBI mice were mostly infiltrated in the injured brain, which initiate the neuro-immune response after TBI, and these infiltrated T cells were effector resident memory T cells (18), while whether these cytotoxic cells resident in the injured mouse brain had a conserved phenotype in human patients needs to be validated in a spatial transcriptomics study.

Regarding the marker genes for cytotoxic immune cells, we found that the expression of CST7 increased in the peripheral blood while the expression of PPP2R2B and cytotoxic profiling decreased. CST7 has been previously reported to increase in Alzheimer’s disease (AD) and as a cytotoxic marker for cytotoxic CD8 T cells in colorectal cancer with a hypomethylation status. We have previously shown that PPP2R2B as a subunit of protein phosphatase 2A had a decreased expression in brain injury animals (TBI model and three epilepsy models) (19, 20). Th17 cells are thought to drive the cytotoxicity of CD8+ T cells in brain insults (21), and increasing levels of IL-17 associated with worse neurological outcomes were found in the peripheral blood through 3 days after onset in stroke patients (22). Activated CD8+ T cells are proven to cause long-term neurological dysfunction in TBI mice (23), and depletion of CD8+ T but not B cells promotes neurological recovery following TBI. Interestingly, a current study from Xiong et al. reported that depletion of B cells is able to reduce the Aβ burden and cognitive impairment in AD mice (24). Although TBI is an independent risk factor for AD, these studies suggest that the heterogeneity of CNS and CD8+ T cells is also a biomarker in AD (13). Although these studies did not mention the source of infiltrated CD8+ T cells in TBI and B cells in AD, it could be predicted that these cells were from the peripheral system. In addition, almost no current studies have linked the immune cells with the coagulation function in brain insults. Based on our findings, it could also be proposed that the expanded peripheral CD8+ T cells might be involved in the progress of PTC. However, the exact role of cytotoxic CD8+ T cells and the neuro-immune interaction needs to be further confirmed in spatial transcriptomic and *in vivo* studies.

Meanwhile, we also showed that the peripheral CD4+ T cells decreased in TBI and correlated with the GCS score as well. To validate this, we first performed WGCNA for the chipset data and identified two modules (MEred and MEturquoise), which are positively and negatively correlated with the GCS score, coagulation dysfunction (Dysf), and Fg value (**Figure S10A, B**). The TOM graph shows that the most interacting modules were MSred and MEturquoise (**Figure S10C**) and the Fg value is mostly correlated with the MSred module (**Figure S10D**). Then, we selected genes from both modules to carry out pathway enrichment analysis and found that the turquoise module was mostly enriched in the T-cell receptor complex, T-cell activation, and lymphocyte differentiation, while the red module was dominantly enriched in neutrophil activation and neutrophil degranulation, which suggests a link between T cells and neutrophils (**Figures S10E, F**). To confirm this, we selected the CD4-related genes in the MEred module and listed both genes (from node in red and to node in blue color, **Figure S10G**). To explore the link between CD4 T cells and neutrophils, we crossed the CD4 nodes, CD4 gene marker, and neutrophil marker and found that only one gene was intersected, named MGST1, as a CD4 node and neutrophil marker, which is not a CD4 T-cell marker (**Figure S10H**). The mRNA expression of MGST1 in human plasma was increased in the severe TBI group compared to the controls (**Figure S10I**, *p* = 0.0161). MGST1 (microsomal glutathione S-transferase 1) is considered to be an antioxidant molecule and has a neuroprotective role in suppressing ferroptosis *via* binding to ALOX5 (25). To validate this, we applied the cell–cell interaction study, and the interaction between ALOX5 and ALOX5AP was only found in the PTC group, but not in the control group (**Figures S10J, K**). In addition, we validated MGST1 as a marker gene for the neutrophil subgroup. From the tSNE analysis, we found that most subsets of neutrophils increased in TBI and PTC compared to the control group (**Figures S11A, B**). MGST1 is a marker gene for subset 8 (**Figure S11C**) and the expression of MGST1 was further validated (**Figures S11D, E**). This might indicate that the interaction between CD4 T cells and neutrophils is involved in the coagulation dysfunction in TBI and needs to be validated in laboratory experiments.

Collectively, a toxic expansion in CD8+ T cells was found to be associated with PTC development. This study only investigated potential biomarkers of PTC progression without a solid experimental validation; however, several immune markers here could be potential targets to reverse the PTC following TBI.

## Data availability statement

The data presented in the study are deposited in the GEO repository, accession number GSE223245 (https://www.ncbi.nlm.nih.gov/geo/query/acc.cgi?acc=GSE223245). The datasets supporting the conclusions of this article are included within the article and **Supplemental Information**. The intersected marker genes for cytotoxic CD8+T, CD4+ T and NK cells are listed as Supple Intersected Markergenes.xlsx. The images with high-resolution were uploaded in the Jianguoyun as: https://www.jianguoyun.com/p/DSqdGXIQu7igCxjYge0EIAA.

## Ethics statement

The studies involving human participants were reviewed and approved by the Ethics Committee of Shanghai Pudong New area People’s Hospital (approval number 2021-K02). The patients/participants provided their written informed consent to participate in this study.

## Author contributions

PZ and BZ participated in the study design. PZ, NZ, and DR collected PBMC samples and did data analyses. CY, QB, and YZ performed the clinical data analyses. PZ and WS wrote the manuscript. All the authors read and approved the final manuscript.
