# Supplementary material for: Integrated single-cell multiomics reveals novel immune candidate markers for post-traumatic coagulopathy
Source: Front Immunol. 2023 Feb 8;13:1095657. doi: 10.3389/fimmu.2022.1095657 (PMC9946684; doi:10.3389/fimmu.2022.1095657)
Supplement: Supplementary file 1 [file DataSheet_1.docx]

**Table S1 Clinical characteristics of patients with traumatic brain injury**

| Patient | Age | Sex | Brain injury | GCS | GCS |
| --- | --- | --- | --- | --- | --- |
| 1. Mi 1 | 48 | F | Laceration | 14 | E3V5M6 |
| 1. Mi 2 | 30 | F | Contusion | 13 | E4V4M5 |
| 1. Mi3 | 40 | M | Contusion | 14 | E4V4M6 |
| 1. Mi4 | 38 | M | SAH | 15 | E4V5M6 |
| 1. Mo1 | 69 | M | Laceration | 11 | E3V3M5 |
| 1. Mo2 | 50 | M | EDH | 11 | E3V4M4 |
| 1. Mo3 | 72 | F | Contusion | 12 | E3V4M5 |
| 1. Mo4 | 29 | M | Contusion | 10 | E2V3M5 |
| 1. Se1 | 39 | F | Brain stem | 4 | E1V1M2 |
| 1. Se2 | 52 | M | SDH | 4 | E1V2M1 |
| 1. Se3 | 38 | M | SDH | 5 | E1V2M2 |
| 1. Se4 | 48 | M | SDH | 4 | E1V1M2 |
| 1. Co1 | 40 | M |  |  |  |
| 1. Co2 | 46 | F |  |  |  |
| 1. Co3 | 50 | F |  |  |  |
| 1. Co4 | 45 | M |  |  |  |

EDH: epidural hemorrhage; SAH：subarachnoid hemorrhage; SDH: subdural hemorrhage;

F: Female, M: Male; E: Eye; V: Verbal; M: Motion

**Table S2 Coagulation parameters of patients with traumatic brain injury**

| **GCS** | **Dysf** | **HB** | **RBC** | **WBC** | **Plt** | **Neutro** | **LN** | **Mono** | **PT** | **INR** | **APTT** | **Fg** |
| --- | --- | --- | --- | --- | --- | --- | --- | --- | --- | --- | --- | --- |
| Co1 | 1 | 152 | 5.07 | 5.19 | 228 | 2.8 | 1.9 | 0.36 | 10.3 | 0.89 | 28 | 1.99 |
| Co2 | 1 | 135 | 4.42 | 5.87 | 274 | 2.59 | 2.6 | 0.52 | 10.3 | 0.89 | 30.8 | 1.93 |
| Co3 | 0 | 142 | 4.82 | 5.48 | 110 | 4.15 | 0.8 | 0.46 | 11.2 | 0.97 | 29.4 | 2.06 |
| Co4 | 0 | 102 | 3.42 | 3.27 | 151 | 2.2 | 0.7 | 0.18 | 11.9 | 1.03 | 28.5 | 2.34 |
| Mi1 | 1 | 110 | 3.56 | 6.86 | 203 | 3.6 | 2.4 | 0.81 | 10.9 | 0.94 | 34.5 | 2.47 |
| Mi2 | 0 | 113 | 3.89 | 11.61 | 260 | 8.41 | 2.3 | 0.85 | 11.8 | 1.02 | 30.7 | 2.52 |
| Mi3 | 0 | 138 | 4.89 | 5.55 | 229 | 2.88 | 1.9 | 0.58 | 10.2 | 0.88 | 27.1 | 2.34 |
| Mi4 | 1 | 111 | 3.77 | 3.85 | 200 | 2.15 | 1.1 | 0.48 | 11 | 0.96 | 33.2 | 2.93 |
| Mo1 | 1 | 129 | 4.6 | 12.74 | 240 | 11.07 | 0.9 | 0.8 | 12.3 | 1.06 | 28.5 | 4.49 |
| Mo2 | 0 | 133 | 4.25 | 9.07 | 248 | 6.57 | 1.7 | 0.78 | 9.9 | 0.85 | 25.1 | 3.68 |
| Mo3 | 1 | 93 | 3.17 | 6.29 | 151 | 4.15 | 1.6 | 0.57 | 10.8 | 0.93 | 30 | 4.68 |
| Mo4 | 0 | 127 | 4.46 | 11.21 | 208 | 10.36 | 0.5 | 0.32 | 13 | 1.12 | 29.8 | 3.49 |
| Se1 | 1 | 103 | 3.43 | 9.87 | 138 | 7.15 | 1.3 | 1.36 | 15.1 | 1.21 | 29.6 | 5.33 |
| Se2 | 1 | 75 | 2.57 | 7.5 | 257 | 5.51 | 1.1 | 0.78 | 10.9 | 0.94 | 32 | 6.13 |
| Se3 | 1 | 93 | 3.15 | 7.15 | 321 | 5.54 | 1 | 0.47 | 11.5 | 0.99 | 27.8 | 4.68 |
| Se4 | 1 | 117 | 4.1 | 5.1 | 60 | 3.6 | 1.2 | 0.27 | 12.9 | 1.11 | 26.2 | 5.33 |

**Table S3 Cell captures by scRNA-seq in 16 samples**

| **Sample id** | **Total_cells_**  **beforeQC** | **Mean_nUMI_**  **afterQC** | **Mean_nGene_**  **afterQC** | **Mean_mito**  **percent_afterQC** | **Total_cells_**  **afterQC** |
| --- | --- | --- | --- | --- | --- |
| Co1 | 18036 | 4026.87 | 1489.87 | 0.04 | 14362 |
| Co2 | 22150 | 3759.17 | 1529.46 | 0.04 | 17866 |
| Co3 | 15030 | 4151.81 | 1642.63 | 0.04 | 12191 |
| Co4 | 17476 | 4240.78 | 1707.57 | 0.04 | 13833 |
| mi1 | 16209 | 3175.90 | 1346.35 | 0.03 | 12764 |
| mi2 | 11892 | 2643.50 | 982.38 | 0.04 | 9521 |
| mi3 | 18301 | 3450.42 | 1300.46 | 0.06 | 14636 |
| mi4 | 13912 | 3778.77 | 1343.37 | 0.05 | 10913 |
| mo1 | 16681 | 5057.45 | 1655.03 | 0.03 | 11637 |
| mo2 | 17442 | 5070.75 | 1746.72 | 0.04 | 14056 |
| mo3 | 23685 | 3698.73 | 1457.15 | 0.03 | 17679 |
| mo4 | 21012 | 3510.99 | 1488.96 | 0.05 | 16957 |
| SE1 | 17467 | 3558.21 | 1324.90 | 0.04 | 12303 |
| SE2 | 7000 | 3251.92 | 1188.60 | 0.03 | 5619 |
| SE3 | 15779 | 5140.88 | 1807.86 | 0.04 | 12666 |
| SE4 | 7074 | 4442.64 | 1500.80 | 0.03 | 5183 |

**
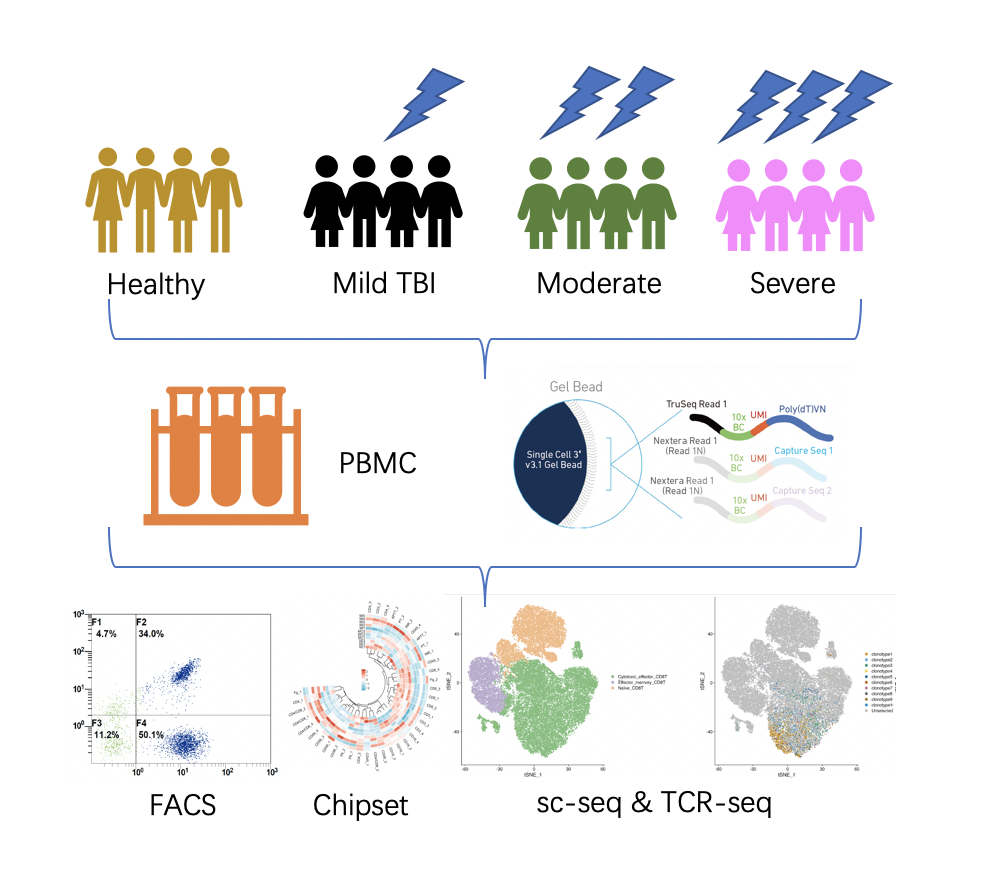
**

**S1.** The schematic plot of the study. After isolating the PBMCs, we applied the 10X scRNA-seq and TCR-seq for these cells and analyzed the results with chipset data.

**
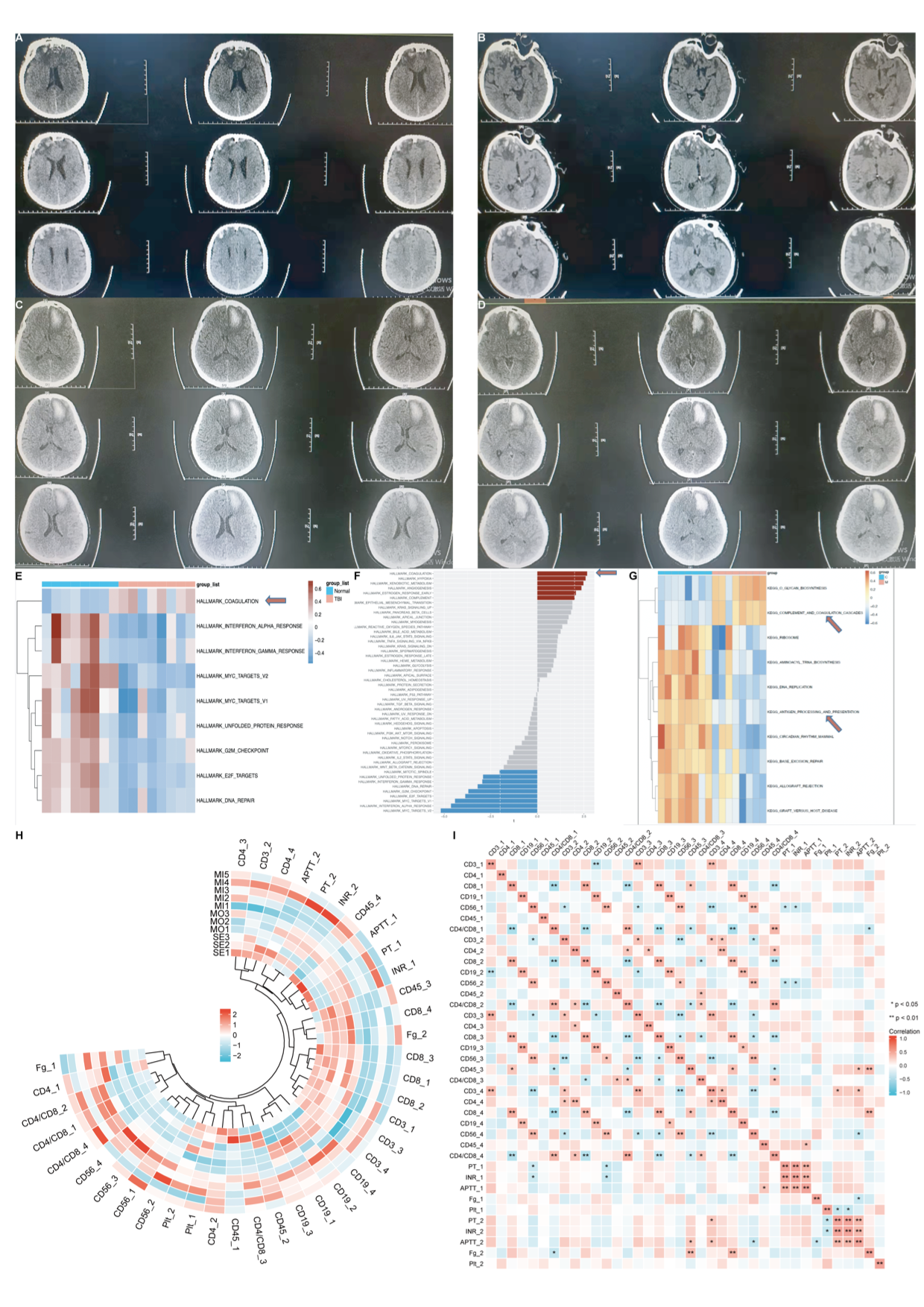
**

**S2. Impaired coagulation function in TBI patients**. (A&B) Day 0 and Day 3 head CT scans show the progressive bilateral frontal contusion; (C&D) Head CT scans show the non-progressive left brain contusion. (E&F) The hallmark enrichments of plasma chipset results for peripheral blood in TBI patients. (G) The KEGG enrichment of plasma chipset results for peripheral blood in TBI patients. (H). The coagulation parameter and immune profiles, we found the increased expression of CD8+ T cells in PBMC with the more severe brain injury. (I) Increased counts of CD8+ T cells were positively associated with the increased Fg value, meanwhile, the counts of NK cells had a negative correlation with the PT and INR value.

**
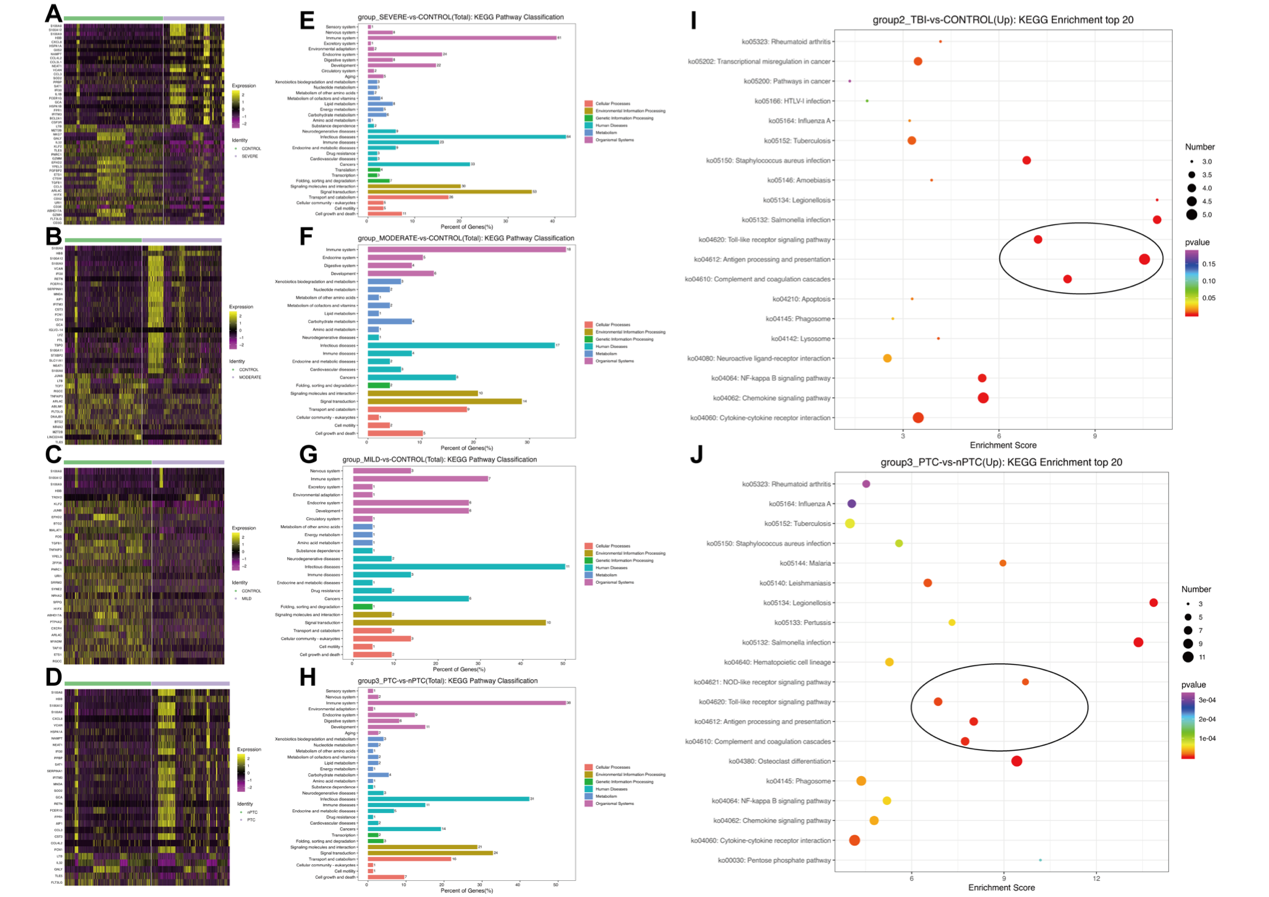
**

**S3. The KEGG enrichment analysis for DEGs in scRNA-seq data.** (A-C) The DEGs between different TBI and control group are listed. (D) The DEGs between PTC and nPTC group. (E-H) KEGG analysis of DEGs identified by single-cell RNA-seq. (I&J) The bubble plots of KEGG analysis of upregulated genes for TBI vs. Control and PTC vs. nPTC. The eclipse indicates the immune and coagulation pathways are involved.

**
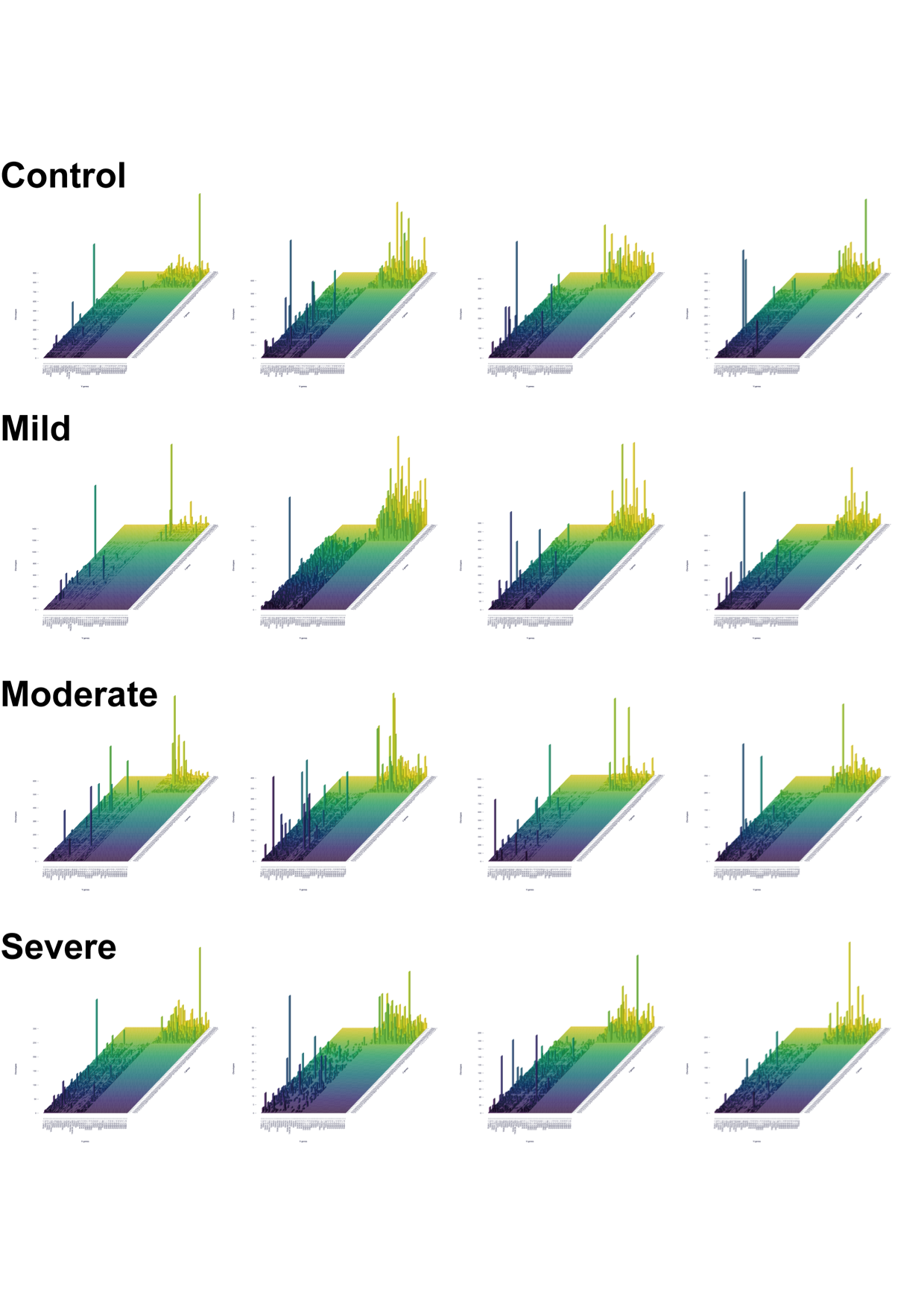
S4. 3D-plot of V-J gene combination.** The clonotypes of V-J gene combinations, X-axis is V gene, Y axis is J gene and Z axis is the number of clonotype.

**
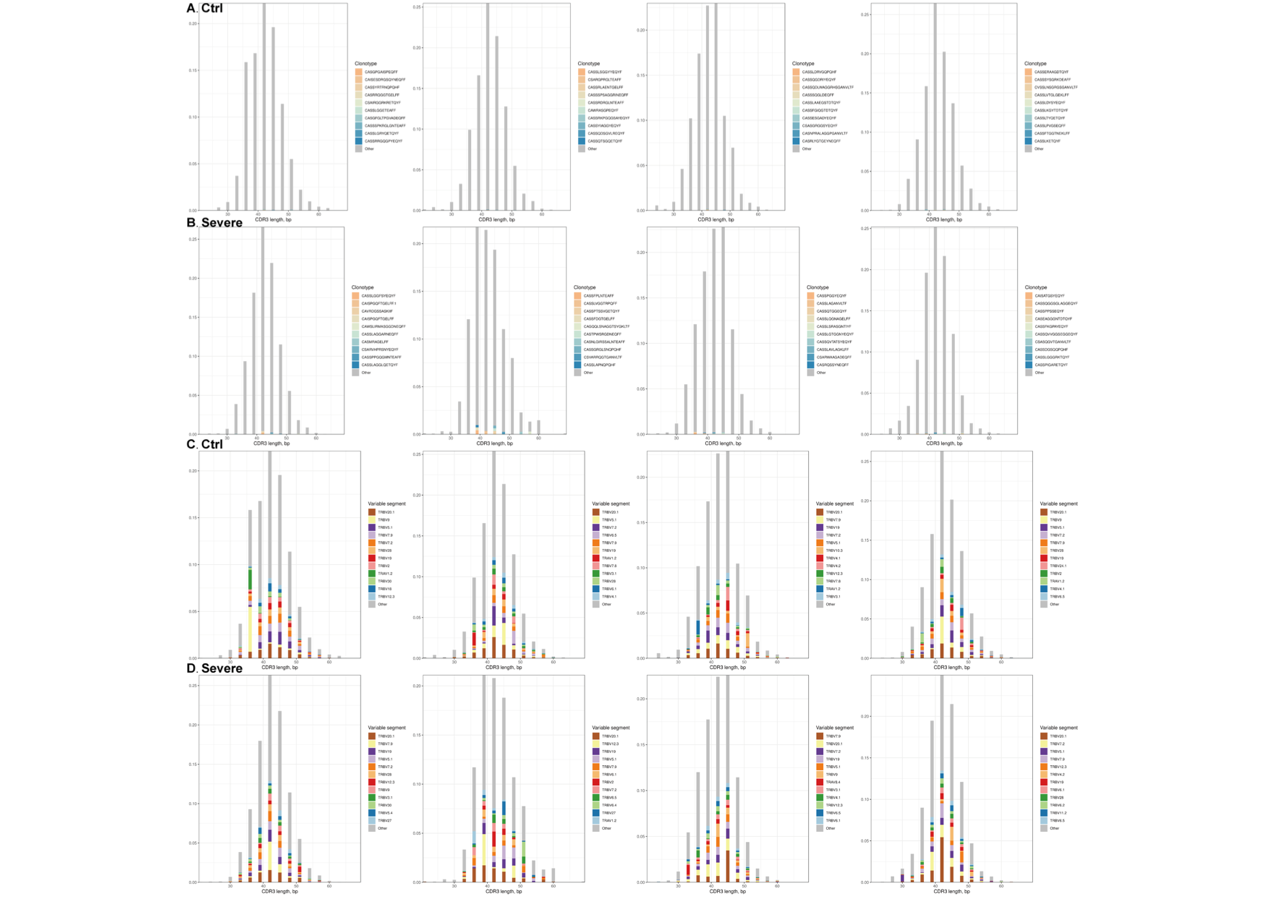
**

**S5. The distribution of different CDR3 length in different samples.** (A&B show the CDR3 increased in severe TBI group); The distribution of V gene in different CDR3 length. The control group shows a symmetry distribution (C) while the severe TBI group demonstrates a bias distribution (D), especially in SE2 and SE3 sample.


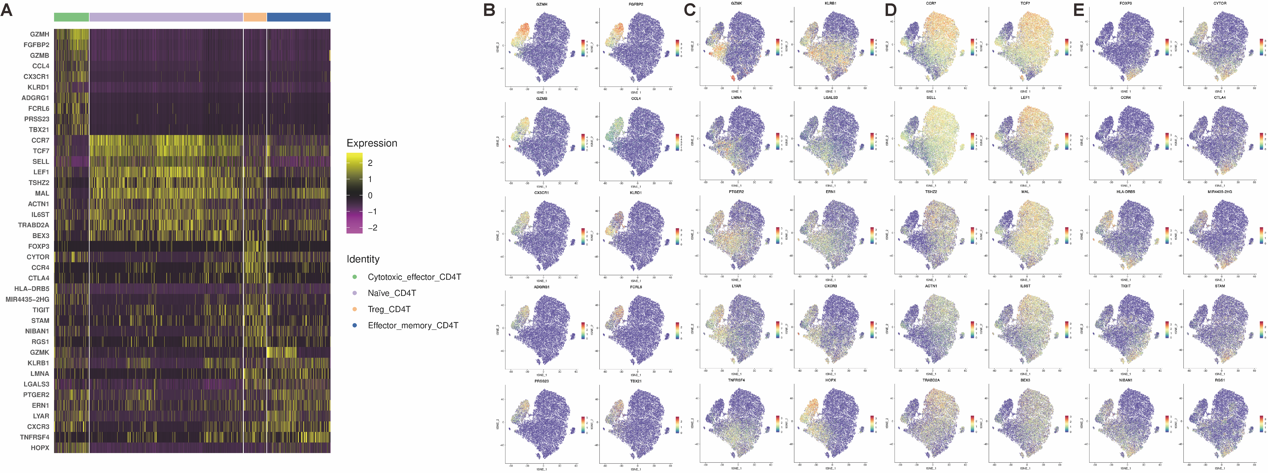


**S6. Molecular markers for CD4 T subclusters.** (A) The heatmap for top 10 molecular markers of CD4 T subcusters. (B) The feature plot for top 10 molecular markers of cytotoxic_effector CD4T. (C) The feature plot for top 10 molecular markers of Naïve CD4T. (D) The feature plot for top 10 molecular markers of Treg_CD4T. (E) The feature plot for top 10 molecular markers of Effector_memory_CD4T.


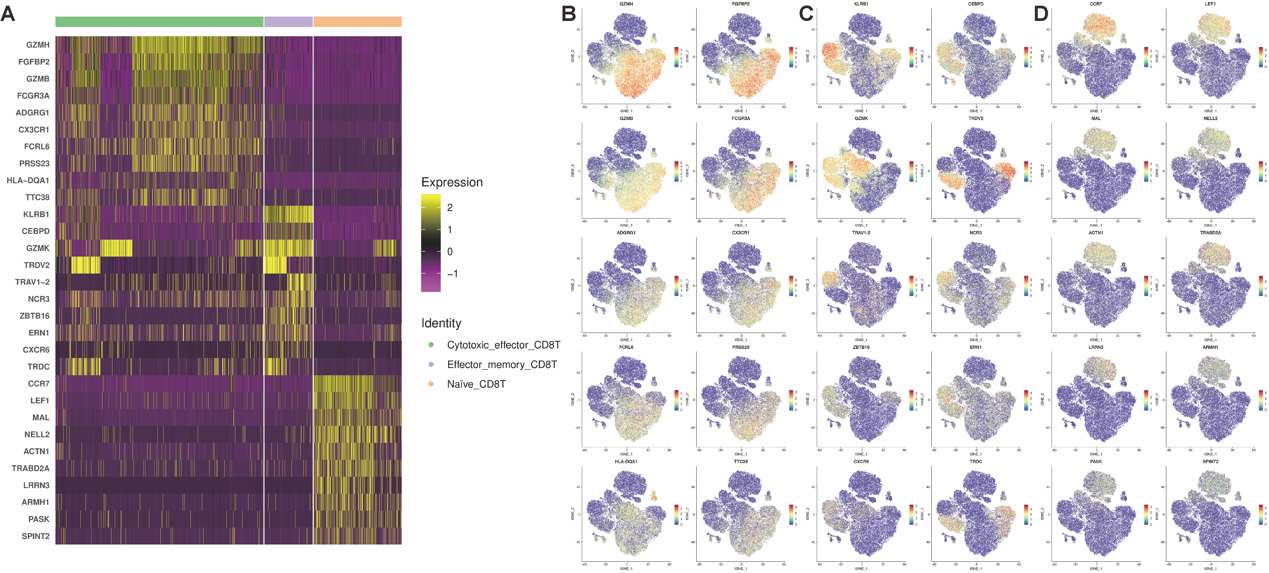


**S7. Molecular markers for CD8 T subclusters.** (A) The heatmap for top 10 molecular markers of CD8 T subclusters. (B) The feature plot for top 10 molecular markers of cytotoxic_effector CD8T. (C) The feature plot for top 10 molecular markers of Effector_memory_CD8T. (D) The feature plot for top 10 molecular markers of Naïve CD8T.


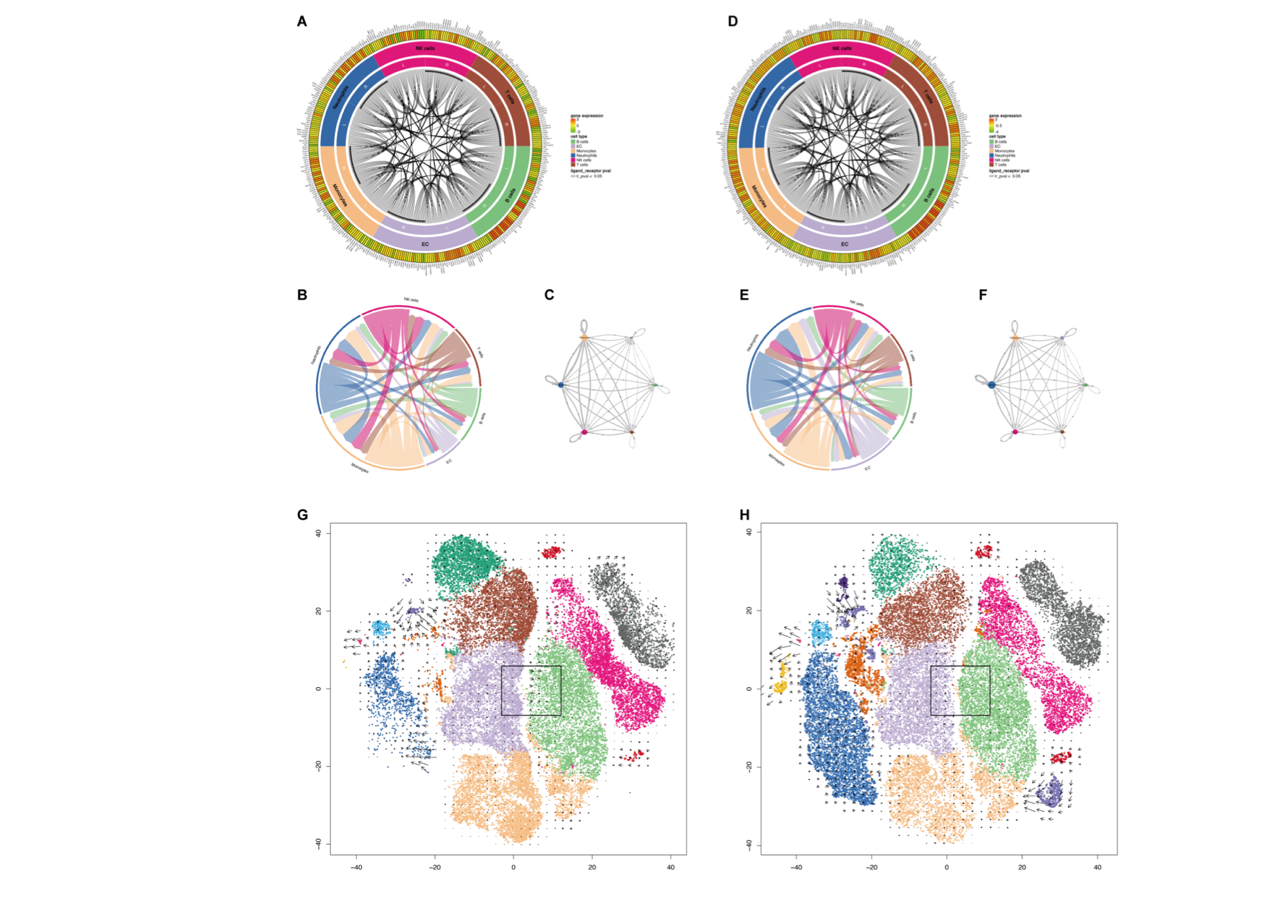


**S8. The cell-cell network and Circos plot between TBI and controls.** (A&D) The outter circle of circos is the ligand/receptor gene expression; The second circle is the cell type and the inner circle is the ligand-receptor gene interaction. The solid line indicates the real interactive effect, while the dot lines not. (B&E) The nodes on the network indicates different cell types and the arrow means the interactive signal from ligand to receptor. The number on the arrow suggests the statistical ligand-receptor interaction. (C&F) This figure also indicates the number of interaction between cells. The outer circle is the cell type, the arrow shows the interactive signal from ligand to receptor cell, the thicker the line, the more the interaction. The RNA velocity in control (G) and TBI (H), which is mapped to the tSNE figure, the different color indicates cell clusters and the arrow suggested the prediction of cell differentiation. The rectangle shows the differentiation of T cell subsets between control and TBIs.


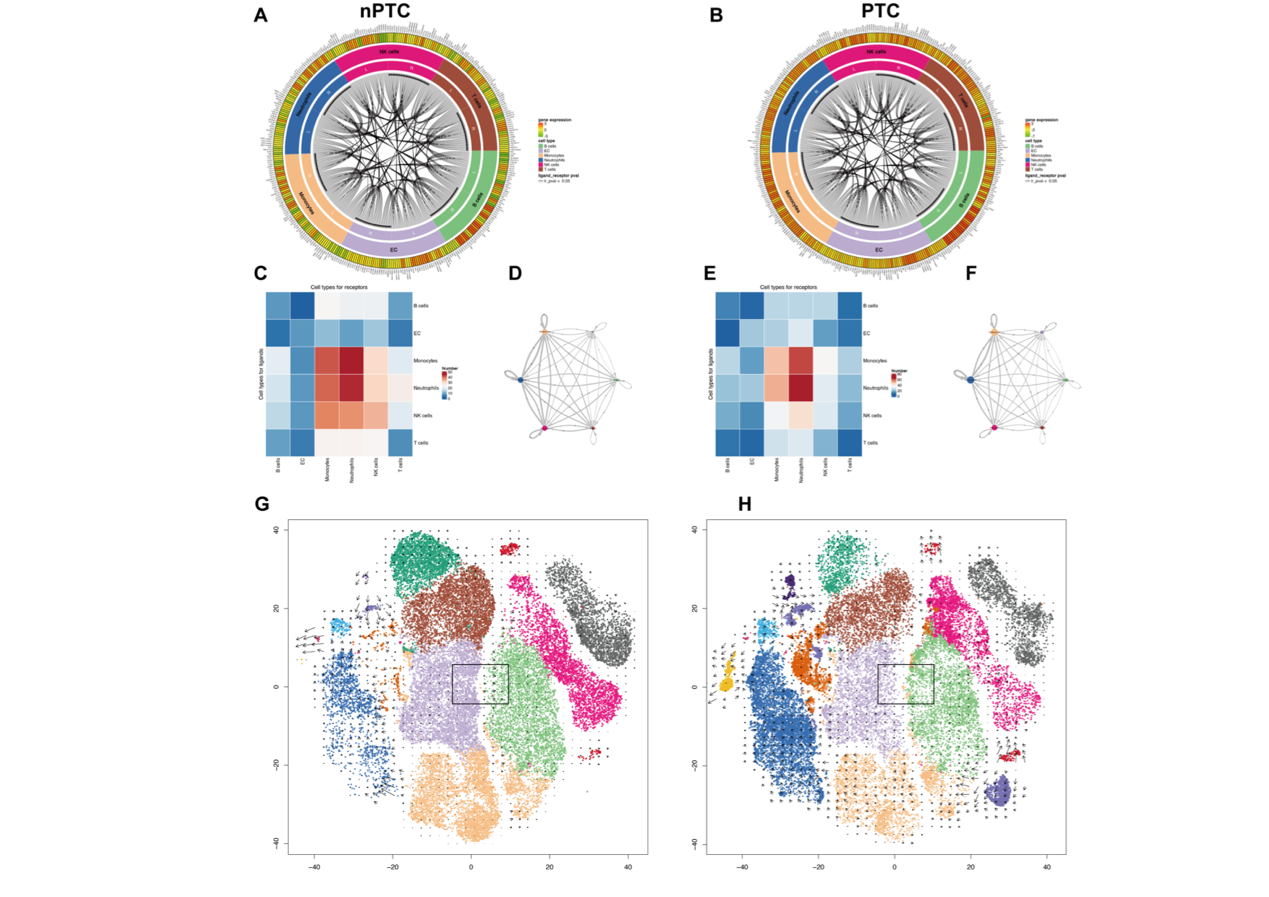


**S9. The cell-cell network and Circos plot between PTC and nPTC.** (A&D) The outer circle of circos is the ligand/receptor gene expression; The second circle is the cell type and the inner circle is the ligand-receptor gene interaction. The solid line indicates the real interactive effect, while the dot lines not. (B&E) The heatmap shows the pair number of interacted cells. The redder, the more pairs. (C&F) This figure also indicates the number of interaction between cells. The outer circle is the cell type, the arrow shows the interactive signal from ligand to receptor cell, the thicker the line, the more the interaction. The RNA velocity in nPTC (G) and PTC (H), which is mapped to the tSNE figure, the different color indicates cell clusters and the arrow suggested the prediction of cell differentiation. The rectangle shows the differentiation of T cell subsets between nPTC and PTC.


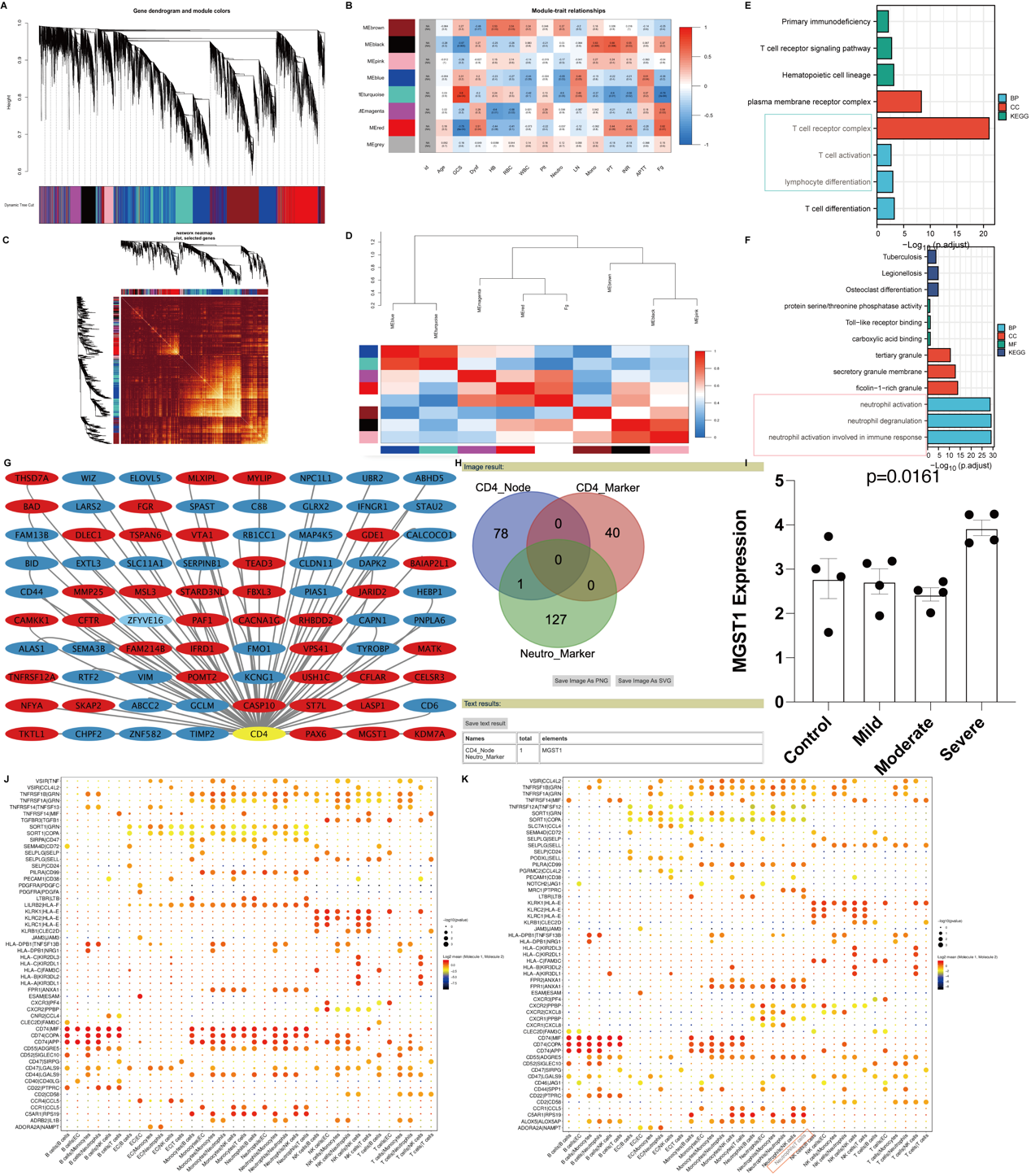


**S10. WGCNA and cell-cell interaction analysis.** A WGCNA analysis shows different gene modules might be involved in TBI. B. The correlation between module genes and phenotypes. C. The TOM figure show the correlation between different modules. D. The cluster of association between module genes and phenotypes. E&F. The pathway enrichmented of module genes (E. MsRed； F：MsTurtoise). G.CD4-related genes in MsRed module. H. The intersected genes among the CD4 marker, neutrophil marker and CD4 node genes. I. The mRNA expression of MGST1 in different groups of TBI. J&K. The dot plot of cell-to-cell interaction in control and PTC group.


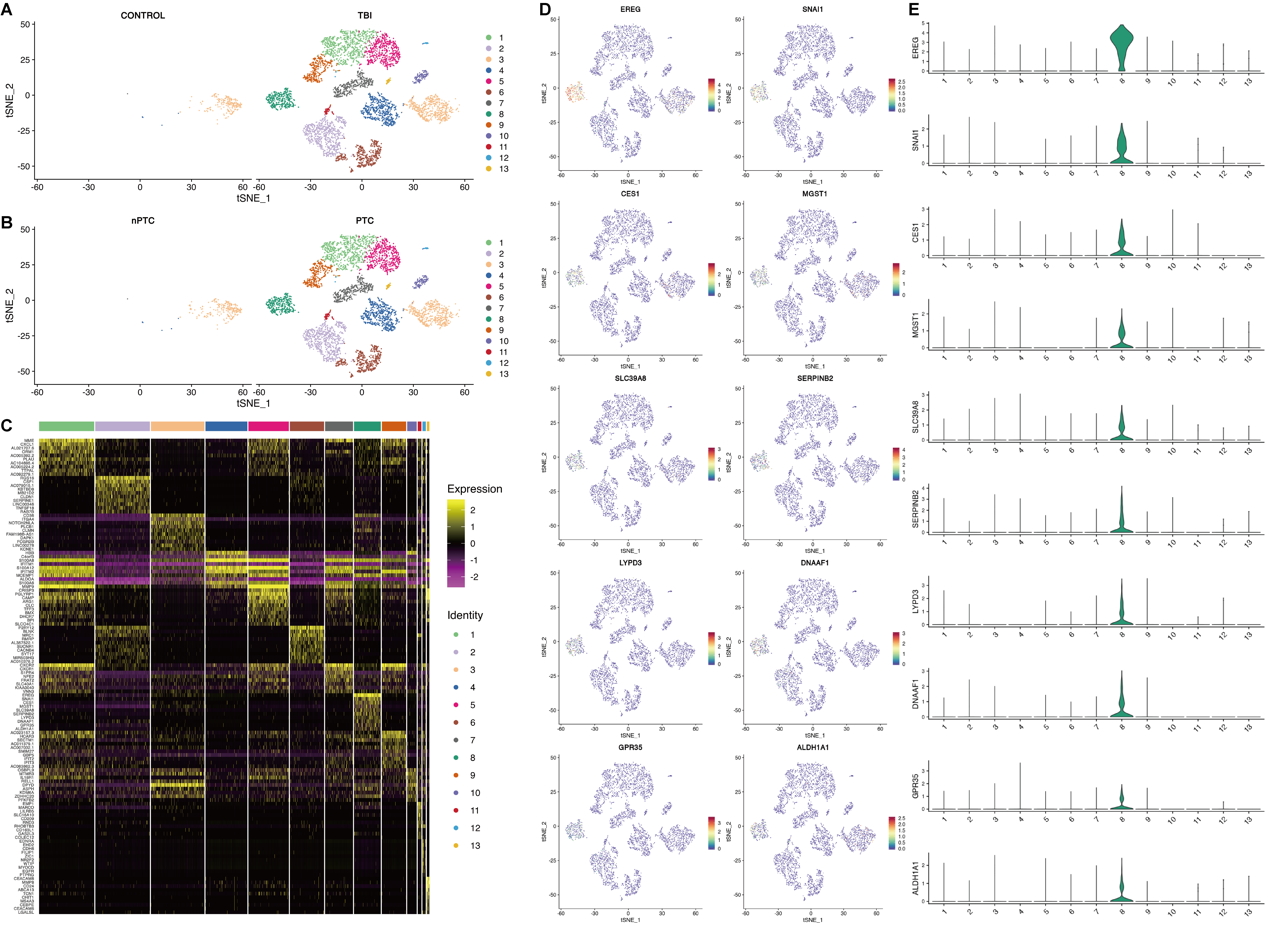


**S11.The subpopulations of neutrophils of PBMCs in TBI patients.** (A) The thirteen clusters of neutrophils between control and TBI. (B) The thirteen clusters of neutrophils between control and PTC. (C) Heatmap shows the marker genes of neutrophil subpopulations. (D&E) Marker genes for neutrophil subset eight with MGST1 listed.
